# Application of Various Machine Learning Techniques in Predicting Total Organic Carbon from Well Logs

**DOI:** 10.1155/2021/7390055

**Published:** 2021-08-30

**Authors:** Osama Siddig, Ahmed Farid Ibrahim, Salaheldin Elkatatny

**Affiliations:** Department of Petroleum Engineering, King Fahd University of Petroleum and Minerals, Dhahran 31261, Box: 5049, Saudi Arabia

## Abstract

Unconventional resources have recently gained a lot of attention, and as a consequence, there has been an increase in research interest in predicting total organic carbon (TOC) as a crucial quality indicator. TOC is commonly measured experimentally; however, due to sampling restrictions, obtaining continuous data on TOC is difficult. Therefore, different empirical correlations for TOC have been presented. However, there are concerns about the generalization and accuracy of these correlations. In this paper, different machine learning (ML) techniques were utilized to develop models that predict TOC from well logs, including formation resistivity (FR), spontaneous potential (SP), sonic transit time (Δ*t*), bulk density (RHOB), neutron porosity (CNP), gamma ray (GR), and spectrum logs of thorium (Th), uranium (Ur), and potassium (K). Over 1250 data points from the Devonian Duvernay shale were utilized to create and validate the model. These datasets were obtained from three wells; the first was used to train the models, while the data sets from the other two wells were utilized to test and validate them. Support vector machine (SVM), random forest (RF), and decision tree (DT) were the ML approaches tested, and their predictions were contrasted with three empirical correlations. Various AI methods' parameters were tested to assure the best possible accuracy in terms of correlation coefficient (*R*) and average absolute percentage error (AAPE) between the actual and predicted TOC. The three ML methods yielded good matches; however, the RF-based model has the best performance. The RF model was able to predict the TOC for the different datasets with *R* values range between 0.93 and 0.99 and AAPE values less than 14%. In terms of average error, the ML-based models outperformed the other three empirical correlations. This study shows the capability and robustness of ML models to predict the total organic carbon from readily available logging data without the need for core analysis or additional well interventions.

## 1. Introduction

Due to the continuous oil and gas exploitation, conventional hydrocarbon reserves are gradually depleted, and the production rates of the current reservoirs are significantly declining.

Conventional hydrocarbon reserves are gradually depleting due to the continuous oil and gas exploitation and the production rates of the current reservoirs dramatically dropped [1, 2]. Source rock and unconventional reservoirs have recently attracted interest as a result [3, 4]. Since unconventional resources are more complex, tight, and less permeable, unconventional reservoirs exploration is more challenging and demanding in contrast to conventional reservoirs [5]. However, considerable discoveries of unconventional resources have been announced around the globe, namely, in North and South America, Middle East, and North Africa, which represents a significant addition to the total world oil reserves [6, 7]. Unconventional resources, in contrast to conventional reservoirs, are self-storing and self-generating reservoirs; consequently, evaluating their hydrocarbon generation potential is critical. Characterization, development, and hydrocarbon extraction of unconventional resources are sophisticated and costly operations, all of which underscores the importance of evaluating the unconventional resources' ability to generate hydrocarbons in a cost-effective and precise manner [4, 6].

Total organic carbon (TOC), which has been widely considered as a quantification of the hydrocarbon generation potentials [8–10], is one of the most efficient parameters that evaluate the quality of unconventional resources [11]. In general, the rock pyrolysis experiment is used to determine TOC content in the laboratory [12, 13]. Due to the high cost of the experiments, there is a limitation on the number of laboratory tests to measure TOC. Consequently, it is very difficult to obtain a complete TOC profile for the formations of the interest, which severely affects the reservoir evaluation [14].

Several authors developed empirical correlations to determine TOC, and these models were developed based on the experimental TOC measures on drilling cuttings or core samples and the corresponding well logs. Then, these developed correlations are proposed to be applied to determine the TOC for different wells [15–19]. These correlations are summarized in Table 1 [20–24]. One concern about these empirical correlations is the low accuracy of the predictions when used with different datasets.

Artificial intelligence (AI) has been used in different industries [25]. AI techniques are known to have the capability to generate high accuracy models; therefore, several studies utilized them in TOC prediction [26, 27]. In the appendix, Table 2 summarizes the different research studies that utilized AI techniques to estimate the TOC from well logs [8, 9, 14, 17, 18, 26, 28–42]. The used well logs include formation resistivity (FR), spontaneous potential (SP), sonic transit time (Δ*t*), bulk density (RHOB), neutron porosity (CNP), gamma ray (GR), and spectrum logs of thorium (Th), potassium (K), and uranium (Ur).

TOC is a vital parameter to characterize the unconventional resources. Experimental analysis can be used to measure the TOC, but it is expensive, time-consuming, and does not give a continuous profile for the total organic carbon with depth. Empirical correlations can be used to estimate TOC. However, there are concerns about the generalization and accuracy of these correlations. In this paper, the application of different AI techniques in TOC prediction in Devonian shale formation from the well logs will be tested. These well logs include sonic transient time, resistivity, bulk gamma ray, bulk density, and spectral GR logs of Th, Ur, and K and neutron log porosity. The next sections in the paper describe the methodology that was used to build the ML models, followed by building the model. The models were then tested and validated with different data sets. A sensitivity analysis was conducted to investigate the importance of input logging parameters in the predicted TOC values.

## 2. Methodology

**Figure 1:**
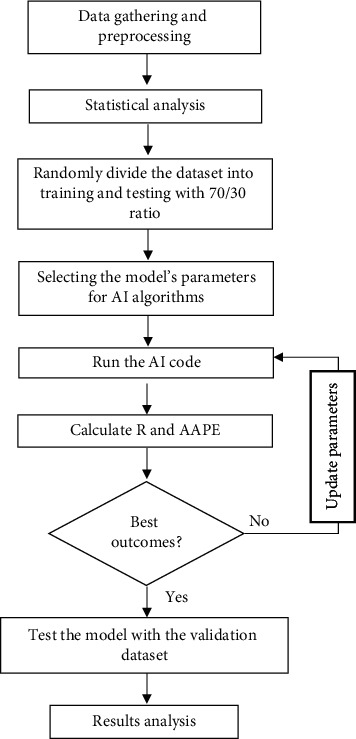
Methodology flow chart.

In this study, three machine learning tools were used to estimate the TOC as a function of eight well logs records.  Figure 1 summarizes the methodology applied for optimizing ML models and validation of these developed models.

### 2.1. Data Description

Experimental data for TOC from three different wells have been collected together with their corresponding well logs. 891, 291, and 82 data points from Well-A, Well-B, and Well-C were used to train, test, and validate the AI models all, respectively. All wells are in Devonian Duvernay shale, which is an organic liquid-rich source rock. The sedimentary basin is located in Alberta, Canada, with 61.7 billion barrels and 443 trillion cubic feet of oil and gas reserves, respectively [43, 44].

### 2.2. Core Samples Testing

**Figure 2:**
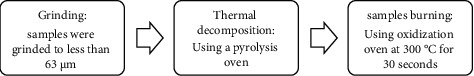
TOC test procedures.

Rock-Eval 6 was employed to estimate the actual TOC values of drilling cuttings from different wells. Tests procedures are shown in Figure 2. More detailed discussion about TOC experimental procedure is presented by Chen et al. [44].

### 2.3. Data Preprocessing

Prior to the AI model's training, outliers, incomplete, or unrealistic data points were eliminated from the data used to construct the model. Data points that contain any value that is away from the mean of the data with three times the standard deviation were considered as an outlier. The statistical characteristics of Well-A's dataset are illustrated in Table 3.

### 2.4. Model Development

The AI model was trained and optimized in this work using Well-A dataset that contains 891 data points with wide ranges of TOC and well logs values. The effect of various parameters inside the AI algorithms was tested to optimize the models, by running the AI tools inside a mutable for loops in MATLAB.

In SVM models, different kernel functions, values for kernel options, epsilon, and regularization were tested while in RF, different sets of number of trees, maximum number of level in each tree, and maximum number of features were used. In DT, various values for maximum tree depth, minimum sample split, and maximum number of features were tested. The correlation coefficient between the known and predicted TOC and the average absolute percentage error (AAPE) were used as evaluation criteria.

In addition, different sets of inputs were used to evaluate the significance of each well log parameter in TOC prediction. Seven sets were considered, the least one includes four parameters, and the most comprehensive consists of all eight parameters, as shown in Table 4.

The accuracies of the generated models were tested and validated using 291 and 82 data points from Well-B and Well-C, respectively. The two wells are in the same field as Well-A. The performance of AI models was also compared with that of currently existing correlations, such as the Schmoker model, the logR approach, and the Zhao et al. model.

## 3. Results and Discussion

The AI models were trained for TOC estimation based on eight well log data of RHOB, Δ*t*, CNL, FR, GR, and spectral GR. The training dataset consisted of 891 data points from Well-A, while the testing dataset contains 291 data points from Well-C. This section presents the results obtained using each method.

### 3.1. Support Vector Machine

**Figure 3:**
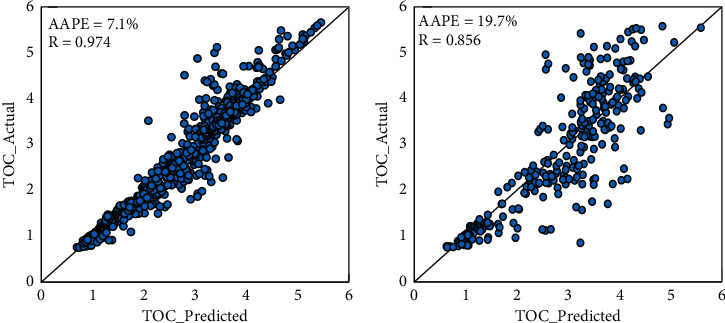
Given and SVM-predicted TOC cross-plots for (a) the training and (b) the testing datasets.

Using data set from Well-A and Well-B, different trials have been applied using support SVM with changing some tuning parameters inside the algorithm, such as kernel function and regularization. The best results were achieved using the Gaussian kernel function. Good results have been achieved in the training dataset with a 7.1% average error; however, the accuracy in the testing datasets was relatively low with an average error of 19.7%. The correlation coefficients were 0.974 and 0.856 for training and testing, respectively.  Figure 3 presents the cross-plots of the actual and SVM-predicted TOC values for the training and testing data sets.

### 3.2. Decision Tree

**Figure 4:**
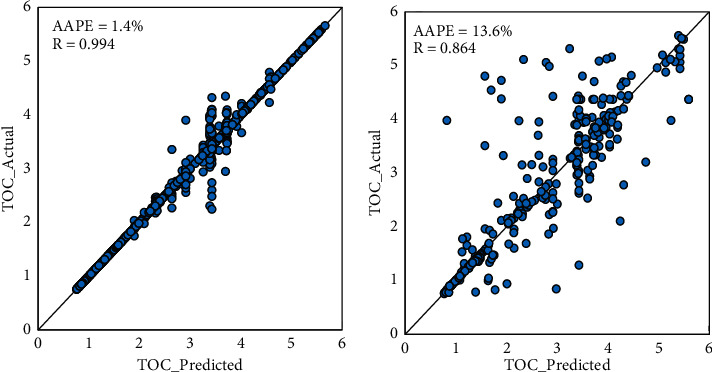
Given and DT-predicted TOC cross-plots for (a) the training and (b) the testing datasets.

This technique resulted in perfect fitting in the training dataset with a 0.994 correlation coefficient as shown in Figure 4(a). However, the prediction performance was significantly less accurate in testing with *R* value which equals 0.864 and which reduces the ability to generalize the model. In Figure 4(b), it is noticeable that some points fall relatively away from the 45° line.

### 3.3. Random Forest

**Figure 5:**
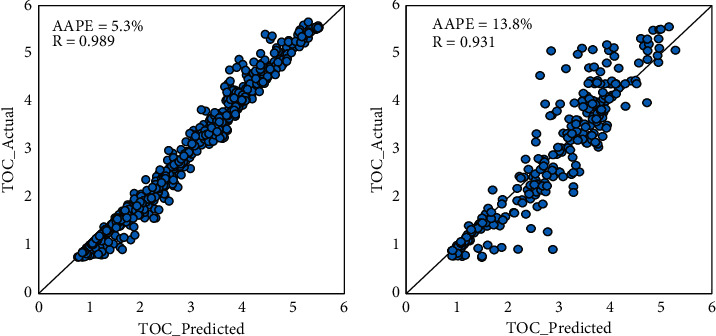
Given and RF-predicted TOC cross-plots for (a) the training and (b) the testing datasets.

In comparison with DT, RF performed better in testing with a 0.931 correlation coefficient, and its performance with the training data set was very close with *R* which equals 0.989.  Figure 5 indicates the deviation between the actual and TOC prediction visually. In comparison with testing results of DT shown in Figure 4(b), it is noticeable in Figure 5(b) that the points fall closer to the 45° line.

## 4. Input Parameters Sensitivity

From the previous analysis, RF was chosen to perform the sensitivity on the inputs. Seven different sets of the well logs information are tested and reported in Table 5. The best performance was achieved when all eight parameters were incorporated (case 7) while the worst fitting accuracy happened when the gamma ray and spectral gamma ray were excluded (case 1). In the cases that exclude density, porosity, and sonic transient time each alone (cases 3, 4, and 6), the least effect in TOC prediction was noticed. It is noteworthy to mention that the effect of changing the inputs' set is more obvious in the testing dataset in contrast to the training dataset.

## 5. Models Validation

As shown in the previous results, good matching accuracies for Devonian shale's TOC have been achieved by the two methods in training and testing datasets. As an additional validation, eighty-two data points from Well-C were kept hidden from the AI tool during model construction utilized later to ensure the generalization of the new models. This well is located in the vicinity of the Well-I and Well-II that has been used in model building. The validation data points fall in 140 ft depth range. The accuracy of the two AI techniques was compared with that of three of the available models for TOC estimation, namely, the Schmoker, Δlog*R*, and Zhao et al. correlations.

**Figure 6:**
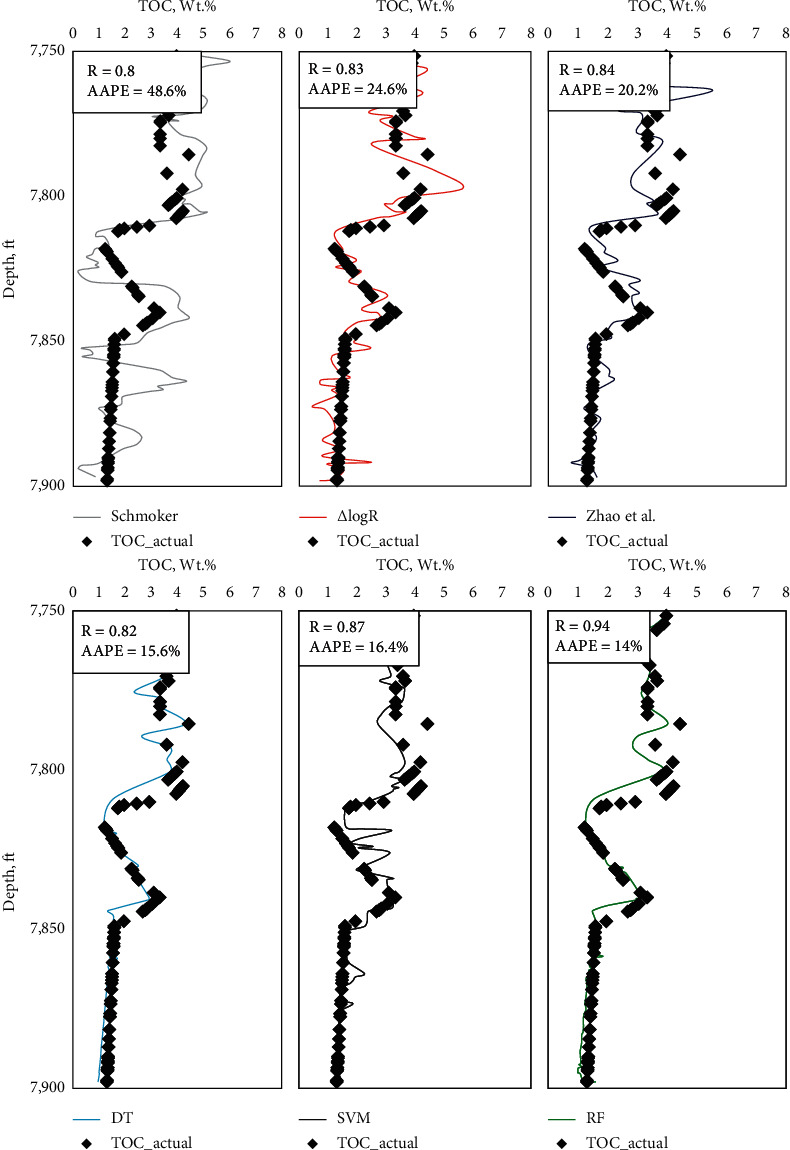
TOC prediction of the AI models and different correlation for the validation dataset of Well-C.

The TOC values obtained from different empirical correlations and AI-based models against the actual values of Well-C dataset are presented in Figure 6. This figure shows that the RF-based model outmatched all other models with AAPE of only 14% and a high *R* of 0.94. DT and SVM models were less accurate than RF; however, in terms of AAPE, both were better than the other three correlations with AAPE values less than 16.4%. Zhao et al. [24] correlation results were the closest to AI-based models and not far from Δlog*R* predictions with AAPE values range between 20% and 25% and *R* values between 083 and 0.83 while the least favorable results in the validation dataset were from the Schmoker model with AAPE above 48%.

The TOC prediction in the different datasets and their comparison with the existing correlations show the accuracy of the developed models for Devonian shale. These models determine the TOC using the conventional well logs and spectral GR logs. It outperformed the existing models that calculate the TOC based on RHOB log only (Schmoker model) or a combination of FR and Δ*t* logs and level of maturity (LOM) (Δlog*R* method) or bulk gamma ray, FR, and Δ*t* or RHOB logs (Zhao et al. [24] correlation). This result and the previous results in this study demonstrate the reliability of the AI models for TOC estimation in Devonian shale.

The developed models were able to accurately predict the TOC from the convention well log including CNP, RHOB, GR, Δ*t*, FR, K, Th, and Ur logs, which helps to obtain a continuous profile for TOC with depth without the need for core analysis or additional well interventions. Similar to any developed model, we recommend employing the developed models using input parameters within the same model's inputs ranges to ensure reliable results. For future work, more data will be collected to validate the developed models and other ML techniques will be applied.

## 6. Conclusions

This study established three models for TOC prediction in Devonian shale from conventional well logs and spectral GR logs using different machine learning techniques and approximately 1250 data points from three wells. The employed ML techniques were support vector machine (SVM), decision tree (DT), and random forest (RF). A summary of the findings reported in this paper is as follows [45]:

- In training and testing datasets, the three AI algorithms produced good matches; however, the RF-based model has the best accuracy. The RF model was able to predict the TOC for the training and testing datasets, with *R* values of 0.99 and 0.93, respectively, and AAPE values of 5.3% and 13.8% in the same order.
- Data from a different well were hidden entirely from the AI tools and used to validate the built model. In this dataset, the RF model produced a 0.94 correlation coefficient and a 14% AAPE.
- The AI-based models' predictions were compared with three other empirical correlations. The AI models yielded more accurate results contrasted to the other models which resulted in AAPEs greater than 20%.

## Figures and Tables

**Table 1:** Summary of different empirical correlations.

Authors	Model	Remarks
Schmoker [20]	TOC(vol. %)=((*ρ*_*B*_ − *ρ*)/1.378) where densities are in g/cm^3^	Predicted TOC in volume percentage and used data from Devonian shale. *ρ* is the formation bulk density, and and *ρ*_*B*_ represents the organic matter free rock density.

Schmoker [21]	TOC(wt. %)=([(100*ρ*_*o*_) − (*ρ* − 0.9922*ρ*_*mi*_ − 0.039)]/[(*Rρ*)(*ρ*_*o*_ − 1.135*ρ*_*mi*_ − 0.675)])	A revised model used Bakken shale's data and predicted TOC in weight percentage. *R* is ratio between the organic matter and organic carbon. *ρ*_*o*_ is the density of the organic matter, and *ρ*_*mi*_ is the average bulk density.

Passy et al. [22]	Δlog*R* =log_10_(FR/FR_baseline_)+0.02 × (Δ*t* − Δ*t*_baseline_) TOC = Δlog*R* ×10^(2.297 − 0.1688 × LOM)^ where resistivity is in ohm·m and transit time is in *μ*s/ft	Widespread model and known as the Δlog*R* model. First, the logs separation (Δlog*R*) is calculated from acoustic transit time (Δ*t*) and formation resistivity (FR). Then, TOC is estimated from Δlog*R* and the level of maturity (LOM).

Wang et al. [23]	TOC =[*α*Δlog*R*+*β*(GR − GR_baseline_)] × 10^(*δ*+*η* *T*_max_)^ where *α*, *β*, *δ*, and *η* are constants	Revised Δlog*R* models to estimate the TOC developed for Devonian shale using Δlog*R*, gammaray (GR), and the indicator of maturity (*T*_max_).

Zhao et al. [24]	TOC =*a*Δlog*R*+*b*(GR − GR_baseline_)+*c* where *a*, *b*, and *c* are constants	Revised Wang's model. Do not depend on the level of maturity, vitrinite reflectance (*R*_*o*_), or *T*_max_.

**Table 2:** Summary of different research studies that employed AI techniques to predict the TOC.

Ref	Data sources	Data points	AI tools	Inputs	Accuracy (*R*^2^)
[8]	Barnett and Devonian shale	442	ANN	RHOB, GR, Δ*t*, FR	0.89–0.93
[9]	Shahejie formation	125	CNN	CNP, RHOB, GR, Δ*t*, FR	0.83
[14]	Kazhdumi formation	31	FL	CNP, RHOB, GR, Δ*t*, FR, K, Th, Ur	0.94
[17]	Devonian and Barnett shales	+500	FNN, SVM	RHOB, GR, Δ*t*, FR	0.74–0.77
[18]	Barnett shale	645	FL	RHOB, GR, Δ*t*, FR	0.91
[25]	Jiumenchong formation	31	SVM	CNP, RHOB, GR, Δ*t*, FR, K, Th, Ur	0.69
[27]	Kangan-Dalan formation	124	FL, NF, NN	CNP, RHOB, GR, Δ*t*, FR	0.85
[28]	Kazhdomi and Kangan-Dalan formations	78	ANN	GR, Δ*t*, FR, K, Th	0.89
[29]	Ordos basin and Canning basin	NA	GPR	CNP, RHOB, GR, Δ*t*, FR, K, Th, Ur	NA
[30]	Gadvan formation	2875	ANN, FL	CNP, RHOB, Δ*t*, FR	0.78–0.99
[31]	Kazhdomi and Kangan-Dalan formations	200	ANN	CNP, GR, Δ*t*, FR, K, Th	NA
[32]	NA	70	ANN	Δ*t*, FR	0.98
[33]	Khatatba and Ras Qattara formations	54	ANN	CNP, RHOB, GR, Δ*t*, FR	0.96
[34]	Beibu Gulf basin	18	SVM	RHOB, GR, Δ*t*, SP, FR	0.75
[35]	Sichuan basin	185	ELM, ANN	CNP, RHOB, GR, Δ*t*, FR, K, Th, Ur	0.87–0.91
[36]	Tonghua basin	215	ANN, SVM	CNP, RHOB, GR, Δ*t*, SP, FR, K, Th, Ur	0.9–0.93
[37]	Barnett and Duvernay shales	460	ANN	RHOB, GR, Δ*t*, FR	0.98
[38]	Barnett shale	442	ANN	RHOB, GR, Δ*t*, FR	0.93
[39]	Barnett shale	+800	ANFIS, FNN, SVM	RHOB, GR, Δ*t*, FR	0.82–0.87
[40]	Qaidam basin	19	ANN	RHOB, GR, Δ*t*, FR	NA
[41]	Bohai Bay, Sichuan, Ordos and western Canada sedimentary basins	353	ANN, SVM	RHOB, GR, Δ*t*, FR	0.89

**Table 3:** The statistical description of the training data.

Statistical parameter	FR (Ω·m)	Δ*t* (*μ*sec/ft)	RHOB (g/cm^3^)	CNP	GR (°API)	Ur (wt%)	Th (ppm)	K (ppm)	TOC (wt%)
Minimum	3.71	51.0	2.39	0.019	22.9	1.39	1.97	0.130	0.76
Maximum	1675	96.6	2.77	0.346	298	22.6	17.0	4.06	5.66
Mean	110	77.9	2.545	0.174	95.5	6.16	9.01	1.51	2.78
Standard deviation	176	8.56	0.075	0.052	38.9	3.16	2.517	0.607	1.30
Kurtosis	21.8	0.227	−0.465	0.984	3.43	6.81	0.315	1.14	−1.08
Skewness	3.98	−0.630	0.436	−0.127	0.837	2.13	−0.135	0.554	0.181

**Table 4:** Different sets of inputs.

No.	Scenarios	Inputs
1	Without GR and spectral GR	CNP, RHOB, Δ*t*,
2	Without spectral GR	CNP, RHOB, GR, Δ*t*, FR
3	Without density	CNP, GR, Δ*t*, FR, K, Th, Ur
4	Without porosity	RHOB, GR, Δ*t*, FR, K, Th, Ur
5	Without resistivity	CNP, RHOB, GR, Δ*t*, K, Th, Ur
6	Without sonic transient time	CNP, RHOB, GR, FR, K, Th, Ur
7	All logs	CNP, RHOB, GR, Δ*t*, FR, K, Th, Ur

**Table 5:** Performance of different sets of inputs.

No.	Inputs	Training		Testing	
		*R*	AAPE (%)	*R*	AAPE (%)
1	CNP, RHOB, Δ*t*,	0.978	8.5	0.811	25.7
2	CNP, RHOB, GR, Δ*t*, FR	0.987	5.8	0.900	16.5
3	CNP, GR, Δ*t*, FR, K, Th, Ur	0.988	5.5	0.924	14.6
4	RHOB, GR, Δ*t*, FR, K, Th, Ur	0.989	5.2	0.921	14.8
5	CNP, RHOB, GR, Δ*t*, K, Th, Ur	0.985	6.5	0.913	16.6
6	CNP, RHOB, GR, FR, K, Th, Ur	0.988	5.5	0.918	14.6
7	CNP, RHOB, GR, Δ*t*, FR, K, Th, Ur	0.989	5.3	0.931	13.8

## Data Availability

Most of the data are included in the manuscript. A detailed data sample will be provided upon request.

## Abbreviations

AAPE: Average absolute percentage error
ANFIS: Adaptive neuro-fuzzy interference system
ANN: Artificial neural network
CNN: Convolutional neural network
CNP: Neutron porosity
DT: Decision tree
ELM: Extreme learning machine
FL: Fuzzy logic
FN: Functional network
FR: Formation resistivity
GPR: Gaussian process regression
GR: Gamma ray
K: Spectrum logs of potassium
ML: Machine learning
NF: Neuro-fuzzy
NN: Neural network
*R*: Correlation coefficient
RF: Random forest
RHOB: Bulk density
SP: Spontaneous potential
SVM: Support vector machine
Th: Spectrum logs of thorium
TOC: Total organic carbo
Ur: Spectrum logs of uranium
*ρ*: Density
Δ*t*: Sonic transit time.
